# Draft Genome Sequence of Enterohemorrhagic *Escherichia coli* O157 NCCP15739, Isolated in the Republic of Korea

**DOI:** 10.1128/genomeA.00522-15

**Published:** 2015-05-28

**Authors:** Taesoo Kwon, Seung-Hak Cho

**Affiliations:** a Division of Biosafety Evaluation and Control, Korea National Institute of Health, Osong, Republic of Korea; b Division of Enteric Bacterial Infections, Center for Infectious Diseases, Korea National Institute of Health, Osong, Republic of Korea

## Abstract

Enterohemorrhagic *Escherichia coli* (EHEC) is the main cause of the recent outbreaks of diarrhea, hemolytic-uremic syndrome (HUS), and hemorrhagic colitis worldwide. Herein, we present the draft genome sequence of the NCCP15739 isolate from a patient in the Republic of Korea.

## GENOME ANNOUNCEMENT

Although most *Escherichia coli* strains in the environment or in the intestinal tract of mammals are nonpathogenic, the following serotypes can cause severe foodborne outbreaks: enterohemorrhagic *Escherichia coli* (EHEC) O26, O103, O104, O111, O118, O121, O145, and O157 (1). The Shiga toxin-producing *E. coli* strain O157:H7 is one of the strains responsible for food poisoning. Previous studies have shown the genome information of *E. coli* O157:H7 EDL933 (2), Sakai (3), TW14349 (4), and EC4115 (5).

Genomic DNA of the NCCP15739 strain isolated from human feces was purified using the whole-genome shotgun protocol for whole-genome shotgun sequencing. The paired-end sequencing library had 500 bp, and the DNA sequencing platform used was Illumina-HiSeq 2000. From whole-genome shotgun sequencing, we generated 555,640,000 reads with 74-fold coverage. The reads were assembled into 479 contigs in 162 scaffolds (*N*_50_ = 146,745) with the SOAPdenovo program (version 1.05) (6), and we determined that the NCCP15739 strain has an approximately 5.3-Mb genome with a 50% G+C content from the assembly. Using Rapid Annotation using Subsystem Technology (RAST) (7) analysis, we identified 5,190 putative open reading frames (ORFs) and 36 RNA genes and can functionally annotate 4,181 (80.6%) ORFs. Functional comparison of the genome sequences available on the RAST server revealed that the *E. coli* O157:H7 EDL933 strain was similar to NCCP15739 (score 520). Comparison of NCCP15739 and EDL933 based on sequence similarity demonstrated that most of the functional genes of NCCP15739 were conserved in *E. coli* O157:H7 EDL933, but 723 genes were unique. The unique genes in NCCP15739, such as the protein secretion system, transcriptional regulator, resistance to antibiotics and toxic compounds, flagella motility, and prophage cluster, explain that phenotypic difference originated due to environmental adaptation. The NCCP15739 strain is related to the enterohemorrhagic human pathogen and produces the Shiga and Shiga toxin-like proteins. It also possesses characteristics such as intimin, invasion, and adhesion and can produce hemolysins in the genome. The genome includes plasmid-borne sequences similar to the plasmid sequence, pSD107, of *Salmonella* enteric substrains. This study will provide information that will enhance the understanding of the O157:H7 type of EHEC, which in turn will aid in the study of the evolution of pathogenic mechanisms through a broader comparative genomic approach of EHEC genomes.

### Nucleotide sequence accession number. 

This whole-genome shotgun project has been deposited in DDBJ/EMBL/GenBank under the accession no. ASHA00000000.
